# Activity of daily life dependency predicts the risk of mortality in patients with COVID-19 undergoing hemodialysis: a retrospective analysis of a single center with nosocomial outbreak

**DOI:** 10.1186/s41100-022-00434-3

**Published:** 2022-09-08

**Authors:** Jun Ino, Fumika Iemura, Chihiro Nakajima, Mio Kodama, Saeko Kumon, Keitaro Sato, Hitoshi Eizumi, Nobuhiro Hijikata, Sadayuki Oshio, Shingo Tachibana, Kosaku Nitta, Junichi Hoshino

**Affiliations:** 1 Department of Nephrology, Toda Central General Hospital, 1-19-3 Hon-cho, Toda City, Saitama 335-0023 Japan; 2 Department of Cardiovascular Medicine, Toda Central General Hospital, 1-19-3 Hon-cho, Toda City, Saitama 335-0023 Japan; 3 Department of Emergency Medicine, Toda Central General Hospital, 1-19-3 Hon-cho, Toda City, Saitama 335-0023 Japan; 4 Department of Surgery, Toda Central General Hospital, 1-19-3 Hon-cho, Toda City, Saitama 335-0023 Japan; 5 Department of Nephrology, Tokyo Women’s Medical University, 8-1 Kawada-cho, Shinjyuku-ku, Tokyo 162-0054 Japan

**Keywords:** COVID-19, Hemodialysis, Activities of daily life dependency, Nosocomial outbreak

## Abstract

**Background:**

We experienced a nosocomial outbreak of coronavirus disease 2019 (COVID-19) from November 2020 to February 2021, during the third wave of the pandemic in Japan.

**Methods:**

We retrospectively assessed the characteristics and data of 20 inpatients undergoing hemodialysis who were hospitalized for treatment of diseases other than COVID-19 during the COVID-19 nosocomial outbreak (“inpatient,” IP), and of 10 outpatients undergoing hemodialysis who were hospitalized for the care of COVID-19 under outpatient visits (“outpatient,” OP).

**Results:**

Eleven patients in the IP group (55%) and one in the OP group (10%) died. Kaplan–Meier analysis showed that the IP group died more rapidly than the OP group (*p* = 0.02). Multivariate analysis among all hemodialysis patients showed that the IP group was not at risk of mortality independently; however, the activity of daily life (ADL) dependency was found to be an independent factor in increasing the risk of mortality (hazard ratio: 7.618).

**Conclusion:**

Our findings show that the nosocomial infected group has a worse prognosis, although it is not an independent predictor for the risk of mortality. ADL dependency could predict the risk of mortality in all hemodialysis patients with COVID-19 during the third wave pandemic in Japan.

## Background

A novel infectious disease, coronavirus disease (COVID-19), caused by severe acute respiratory syndrome coronavirus 2 (SARS-CoV-2), has spread worldwide and threatens human health as well as the public medical system. The mortality rates differ not only between severities (35% in critical grade [1] and 2% in overall grade [2]) but also between races, regions, or nations, among hospitalized patients (20.3% in the US [3], 28% in China [4], and 7.5% in Japan [5]). A systematic review has identified pre-existing comorbidities, such as hypertension, diabetes, cardiovascular diseases (CVD), and chronic kidney disease (CKD), as risk factors associated with severity and mortality rate among patients with COVID-19 [6]. In addition, in Japan, it has been reported that patients with several exacerbating factors for this infection, including aging, obesity, hypertension, hyperuricemia, chronic obstructive pulmonary disease, and CKD, are more likely to develop severe illness and die during the clinical course [1, 7, 8]. Epidemic reports on patients undergoing hemodialysis suggest that aging and potential immunodeficiency worsen the prognosis of various infectious diseases [9, 10], and studies indicate that the mortality rate of dialysis patients with COVID-19 is higher than that of non-dialysis individuals with COVID-19 in various countries [11–15].

Nosocomial outbreaks of COVID-19 occur in medical and care facilities worldwide owing to the strong infectious potential of SARS-CoV-2. A study showed that the mortality rate of non-dialysis inpatients with COVID-19 was 4.3% during a COVID-19 outbreak in the hospital [16]. However, no study has investigated the mortality rate of dialysis inpatients during a nosocomial outbreak. In the third wave of COVID-19 in winter 2020–2021, a nosocomial outbreak of COVID-19 occurred in our hospital, and the function as a general hospital for accepting emergency patients and caring for new inpatients was frozen at all clinical departments, except for outpatient treatment in the dialysis unit. This study aimed to compare the survival rate between inpatients and outpatients with COVID-19 undergoing hemodialysis, and to determine the potential risk for COVID-19-induced mortality in the inpatient group. Moreover, we analyzed potential factors causing the nosocomial outbreak of COVID-19 by reviewing strategies for infection control used in our dialysis unit.

## Methods

### Study design and participants

Our hospital experienced a nosocomial outbreak of COVID-19 for approximately three months, from November 2020 to February 2021. During this period, we identified positive results for COVID-19 testing in hemodialysis patients during hospitalization for diseases other than COVID-19, and in those with outpatient visits. We retrospectively assessed the characteristics of 30 hemodialysis patients with COVID-19 between December 2020 and March 2021. In this retrospective cohort study, we compared the mortality rate between the nosocomial inpatients and outpatients. We further investigated the risk of nosocomial infection for mortality in hemodialysis patients with COVID-19.

All procedures were conducted according to the ethical standards of the institutional review board of our hospital (IRB approval number 0506), and the 1964 Declaration of Helsinki and its later amendments. Although the retrospective analysis using anonymous clinical data did not require written patient consent, we obtained informed consent in the form of an opt-out notice after presenting the information of this study on the website of our hospital.

### Overview of the nosocomial outbreak

Our hospital has 517 beds, including 10 in the intensive care unit (ICU) and 6 in the cardiovascular care unit, and 26 major medical departments, except the infectious disease department. The dialysis unit has a total of 30 beds, including one private room, where hemodialysis therapy is performed for approximately 35–40 outpatients and 10–20 inpatients daily.

Since the nationwide spread of COVID-19 in Japan in February 2020, in our dialysis unit, we performed a practical infection control against COVID-19 according to the guidance published by the COVID-19 Task Force Committee of the Japanese Association of Dialysis Physicians, the Japanese Society for Dialysis Therapy, and the Japanese Society of Nephrology [11].

The nosocomial outbreak of COVID-19 in our hospital began on November 15, 2020, coinciding with the third wave of pandemics, and to limit the function of the hospital, we suspended medical care for all new hospitalizations, including outpatients undergoing hemodialysis who were eligible for hospitalization. The definition of nosocomial infection of COVID-19 was as follows: (1) patients hospitalized after November 15, 2020, who were positive for COVID-19 testing (excluding patients who were positive more than two weeks after discharge), (2) hemodialysis outpatients from November 15, 2020 onward, who were positive for COVID-19 testing, and (3) medical workers who have been working in our hospital since November 15, 2020 and tested positive for COVID-19. The COVID-19 test was used to screen all patients and medical workers in the ward where the infected case occurred, as well as symptomatic inpatients, medical workers, or close contacts in any wards.

The index case in the hemodialysis unit was identified on December 16, 2020. The patient was hospitalized in the nephrology department ward, which was recognized as the second nosocomial infection site. Within a few days, the number of infected inpatients increased in the dialysis unit, and simultaneously, infected individuals were confirmed one after another in each ward. Eventually, a nosocomial infection spread throughout the hospital. After 15 positive dialysis inpatients were identified within seven days of the index case in the dialysis unit, the first positive dialysis outpatient was identified in the dialysis unit. Ten days later, one medical worker in the dialysis unit was identified as being positive for COVID-19. In total, we confirmed positive results for COVID-19 testing in 22 dialysis inpatients, 6 dialysis outpatients, and 1 medical worker in the dialysis unit, during the period of the nosocomial outbreak. A new positive case with COVID-19 testing in the dialysis unit was not confirmed after January 11, 2021. Under the guidance of the COVID-19 cluster group of the Ministry of Health, Labor, and Welfare, which was dispatched to our hospital on January 6, 2021, at the request of the prefecture, we investigated the causes of the rapid and widespread nosocomial outbreak of COVID-19 and then took thorough measures against each of these causes. After confirming that there were no new cases of COVID-19 for two weeks in the hospital, we issued the end declaration of the nosocomial outbreak of COVID-19 on February 26, 2021. After this declaration, by March 2021, we accepted four new dialysis outpatients who were infected via community-onset, and they were enrolled as the OP group. At the time of submission of this paper, we experienced no other nosocomial outbreaks of COVID-19 in the dialysis unit.

### Dialysis patients with COVID-19 during hospitalization

In the nosocomial outbreak, we encountered 22 inpatients undergoing hemodialysis under care for other than COVID-19 (“inpatient,” IP). The details of the inpatient departments and hospitalization reasons were as follows: 12 in nephrology (hemodialysis initiation, 7; CKD with infectious diseases other than COVID-19, 3; CKD with appetite loss, 1; and examination of urine abnormalities, 1), three in cardiovascular medicine (one each in peripheral arterial disease, loss of consciousness, and aortic dissection), three in neurology (cerebral infarction, 2; chronic subdural hematoma, 1), two in orthopedics (fracture of the femur neck, 1; fracture of the ilium, 1), one in gastroenterology (colon polypectomy), and one in dermatology (lower limb ulcer due to diabetic gangrene). Because there was no isolated private space for a dialysis session for each inpatient, eight infected hemodialysis inpatients were transferred to another hospital that accepted COVID-19 cases, and we continued to treat 14 inpatients in the IP group in our hospital without being able to be transferred.

### Dialysis patients with COVID-19 during outpatient visits

Until the end declaration of the nosocomial COVID-19 outbreak, we suspended new hospitalization care for all outpatients: referral patients and regular outpatient visits, including dialysis unit visits. During this period, six hemodialysis outpatients were tested positive for COVID-19: five patients were transferred to hospitals that accepted patients with COVID-19, and one was hospitalized in our hospital without being able to be transferred. After the end declaration on February 2021, we initiated a new hospitalization treatment for hemodialysis patients with COVID-19, including one visiting our dialysis unit and three being referred from another hemodialysis clinic until March 2021. None of these four outpatients, who were admitted after the start of the domestic supply of the COVID-19 vaccine (February 2021), had received the vaccine; one patient was hospitalized in another hospital during the relevant period, and three lived in a municipality where the vaccine supply had not started at the time of their admission. Finally, we analyzed 10 hemodialysis outpatients hospitalized for COVID-19 (“outpatient,” OP).

### Diagnosis of COVID-19, classification of the severity grade, and treatment for COVID-19

All patients were diagnosed with SARS-CoV-2 infection in nasopharyngeal samples using reverse transcriptase-polymerase chain reaction or quantitative antigen test. The day of onset was the day when symptoms that were characteristic of COVID-19, such as fever of ≥ 37.5 °C, respiratory symptoms, or taste and olfactory dysfunction, appeared, or the day when the COVID-19 test, containing one for screening purposes, was first identified to be positive.

We used a severity classification that is defined in the clinical management of patients with COVID-19, published on the website https://www.mhlw.go.jp/content/000712473.pdf. In summary, there are four categories of severity based on peripheral oxygen saturation (SpO2) and clinical states: mild, SpO2 ≥ 96% and no respiratory symptoms; moderate I (Patients do not suffer respiratory failure), 93% < SpO2 < 96% and shortness of breath and pneumonia findings; moderate II (patient suffering respiratory failure), SpO2 ≤ 93% and oxygen administration required; and severe, admission to the ICU or mechanical ventilator required. On hospitalization or identifying positive for COVID-19 testing, no hemodialysis patients in both the IP and OP groups were diagnosed as being severe grade, based on the above criteria: 13 patients had mild and 7 patients had moderate II grade in the IP group, whereas 9 patients had mild and 1 patient had moderate II grade in the OP group.

The selection of treatments for hemodialysis patients with COVID-19 depended on the attending doctors of the department or medical institutions providing inpatient treatments.

### Parameters and study outcomes

We investigated backgrounds, vital signs, and blood samples at the time of COVID-19 onset, as well as the final prognosis of all infected hemodialysis patients. The following information was collected from in-hospital electronic medical records of patients in our hospital, or from the clinical course stated in the referral letters of patients transferred to other facilities: (1) the symptoms and vital signs during diagnosis of COVID-19, as well as serological parameters related to the degree of inflammation and risk background for mortality in hemodialysis patients with COVID-19; (2) treatments with drug medications, such as antiviral agents and steroids; (3) day of one-stage progression of severity, day of development to the severe grade, and day of mortality, which were defined as study outcomes. CVD history included a history of ischemic heart disease, congestive heart failure requiring admission, cerebral vessel disease, and peripheral arterial disease. As a parameter of activities of daily life (ADL) dependency, the wheelchair user or certified caregiver was determined based on the following definition: (1) those who needed assistance from others in daily life or in the medical setting, even before they developed COVID-19 infection; (2) those who had physical or mental declines in mobility that prevented them from walking safely on their own; and (3) those who required gradual intensity of caregiving due to decreased ADL. In addition, patients who met the definition of either wheelchair users or certified caregivers were scored using the Clinical Frailty Score (CFS) as an objective measure [17].

### Statistical analyses

To test the difference between the IP and OP groups, the Mann–Whitney *U *test was performed to analyze continuous variables, and Fisher’s exact test was performed to analyze nominal variables. To evaluate whether nosocomial outbreaks had worsened the severity and mortality, we constructed a Kaplan–Meier curve for each outcome and performed the log-rank statistical analysis. To identify whether nosocomial COVID-19 increases the risk for mortality in hemodialysis patients, we performed the Cox proportional hazards regression analysis, including univariate, followed by multivariate analysis, and examined the hazard ratio (HR) and 95% confidence interval (CI) for the death-censored endpoint. We constructed a clinical prediction model by sequentially introducing our target variates (IP group) and other major variables into the Cox proportional hazard model. In the Cox model 0, the HR was an unadjusted value of univariate analysis by the IP group for the endpoint. In model 1, the HR was adjusted for possible major confounders, age and sex, and in model 2, the HR was adjusted for the aforementioned confounders in addition to variables based on subject matter knowledge identified in previous reports: laboratory parameters [serum albumin and C-reactive protein (CRP) levels at the time of COVID-19 diagnosis] and patients’ characteristics (ADL dependency at the onset of COVID-19).

In Table 1, the values of continuous variables are presented as the median and interquartile ranges in parentheses, whereas nominal variables are presented as the number of cases and percentages in parentheses. Differences were considered significant at two-tailed *p* values < 0.05 in all analyses. Statistical analyses were performed using the statistical software EZR (Saitama Medical Center, Jichi Medical University, Saitama, Japan).

**Table 1:** Baseline characteristics and demographics of patients

	Overall (n = 30)	Outpatients (n = 10)	Inpatients (n = 20)	*p* value
Age (year)	57.5 (57.3–79.8)	57.5 (41.5–65.8)	76.5 (67.0–81.0)	0.006
Male/female	23/7	7/3	16/4	0.657
Duration of dialysis (months)	54.0 (2–92.8)	91.5 (50–107)	29.0 (1–180)	0.077
DM history	18 (60.0)	5 (50.0)	13 (65.0)	0.461
CVD history	22 (73.3)	7 (70.0)	15 (75.0)	1.000
Wheelchair user or certified caregiver	9 (30.0)	1 (10.0)	8 (40.0)	0.204
Clinical frailty score				0.571
Score 7	7	1 (10.0)	6 (30.0)	
Score 8	2	0 (0.0)	2 (10.0)	
Current smoking	2 (6.7)	1 (10.0)	1 (5.0)	1.000
Past smoking	21 (70.0)	9 (90.0)	12 (60.0)	0.204
Symptoms on onset				0.172
Fever	14 (46.7)	10 (100.0)	9 (45.0)	
Cough	2 (6.7)	1 (10.0)	1 (5.0)	
Sore throat	1 (3.3)	0 (0.0)	1 (5.0)	
Taste dysfunction	2 (6.7)	1 (10.0)	1 (5.0)	
Short of breath	1 (3.3)	0 (0.0)	1 (5.0)	
Diarrhea	1 (3.3)	0 (0.0)	1 (5.0)	
Positive by screening testing	6 (20.0)	0 (0.0)	6 (30.0)	
COVID-19 severity grade				0.459
Mild	22 (73.3)	9 (90.0)	13 (65.0)	
Moderate	8 (26.7)	1 (10.0)	7 (35.0)	
Severe	0 (0.0)	0 (0.0)	0 (0.0)	
Systolic blood pressure (mmHg)	135.5 (120.0–157.8)	149.0 (132.3–173.5)	129.0 (115.5–147.3)	0.172
Diastolic blood pressure (mmHg)	71.0 (62.5–83.5)	78.5 (64.5–105.0)	67.5 (63.5–80.3)	0.179
Heart rate (beat/min)	81.0 (76.5–94.5)	98.5 (67.8–104.8)	79.5 (70.8–85.5)	0.007
Respiratory rate (/min)	18 (16–19.5)	19 (18–20)	16 (16–18)	0.158
SpO2 (%)	96.0 (95.3–98.0)	96.5 (95.3–97.0)	96.0 (95.8–98.0)	0.621
Body temperature (degrees)	38.0 (37.3–38.3)	38.2 (38.0–38.4)	37.5 (37.0–38.2)	0.048
Body mass index (kg/m^2^)	23.1 (21.3–26.7)	25.8 (23.3–29.1)	22.3 (20.5—25.5)	0.100
Antiviral agent (favipiravir)	3 (10.0)	3 (30.0)	0 (0.0)	0.030
Steroids (dexamethasone)	21 (70.0)	9 (90.0)	12 (60.0)	0.204
White blood cell (/μL)	6935 (6115–9160)	7025 (5022–7890)	6710 (6125–9632)	0.660
Lymphocyte (/μL)	772 (589–1006)	872 (769–1000)	638 (552–1063)	0.143
Hemoglobin (g/dL)	10.5 (9.7–11.5)	11.4 (10.7–12.3)	10.0 (9.2–11.1)	0.013
Albumin (g/dL)	3.10 (2.7–3.68)	3.75 (3.45–3.98)	2.85 (2.50–3.20)	0.000
LDH (mg/dL)	255.5 (198.8–319.0)	239.5 (186.3–359.0)	256.5 (206.3–311.0)	0.588
Ferritin (ng/mL)	141.2 (72.0–208.0)	89.5 (43.0–144.1)	150.6 (110.8–253.9)	0.109
CRP (mg/dL)	2.15 (0.86–5.68)	2.59 (1.96–3.45)	1.82 (0.82–6.44)	0.826
Complication of other infections	13 (43.3)	2 (20.0)	11 (55.0)	0.119

Continuous variables are presented as median (interquartile range), and nominal variables are presented as numbers (%). *CI* confidence interval, *CRP* C-reactive protein, *CVD* cardiovascular disease, *DM* diabetes mellitus, *LDH* lactate dehydrogenase isozyme, *SpO2* peripheral oxygen saturation

## Results

### Analysis of clinical outcomes

**Fig. 1:**
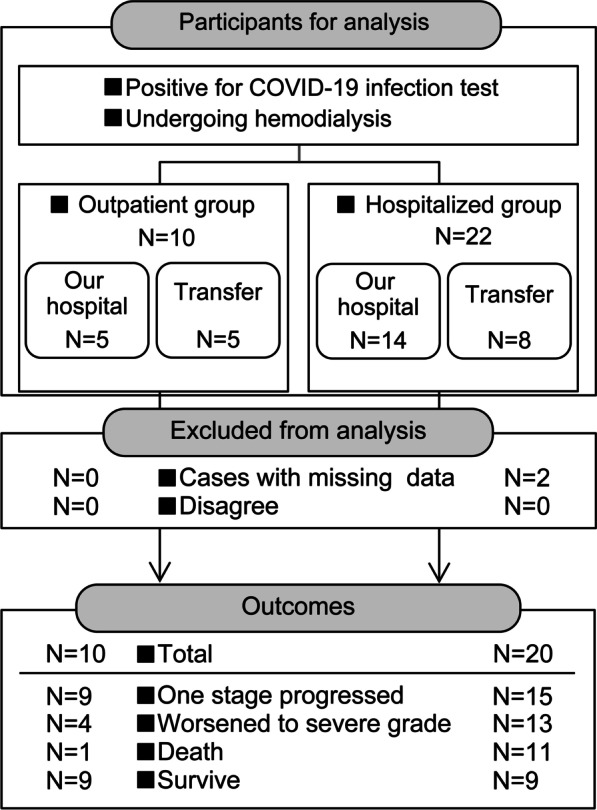
Flowchart of the patient selection process and main outcomes. COVID-19, coronavirus disease 2019

The study population comprised 10 outpatients and 20 inpatients with diagnoses of COVID-19 undergoing hemodialysis after excluding two inpatients because of no available information from the transfer facility (Fig. 1). All 30 patients were followed until death or were censored at discharge. Five patients in the OP group and six in the IP group were transferred to other hospitals, and five in the OP group and 14 in the IP group were treated in our hospital. During the clinical course, nine patients in the OP group and 15 in the IP group showed one-grade progression, four in the OP group and 13 in the IP group worsened to severe grade, and one in the OP group and 11 in the IP group died of COVID-19 or related commodities. One patient who died of related commodities had chronic heart failure, and the other had bacterial pneumonia. As a result, nine patients (90%) in the OP group and nine (45%) in the IP group recovered and were discharged. All 18 discharged patients were confirmed to be alive at least six months after discharge (mean observation period 13.9 ± 4.7 months).

Patient characteristics and laboratory data at the onset of COVID-19 are presented in Table 1. Compared with the OP group, the IP group had older age (IP vs. OP: 73.8 ± 13.6 vs. 54.6 ± 17.6 years), lower serum albumin (IP vs. OP: 2.83 ± 0.51 vs. 3.70 ± 0.39 g/dL), and lower hemoglobin levels (IP vs. OP: 10.2 ± 1.6 vs. 11.8 ± 1.9 g/dL). The IP group also showed lower heart rate and body temperature than the OP group (heart rate: 78.0 ± 10.5 vs. 94.4 ± 18.9 beat/min; body temperature: 37.6 ± 0.9 vs. 38.3 ± 0.5 °C). No significant difference was found between the two groups in an initial grade of severity (13 mild and 7 moderate grades in IP vs. 9 mild and 1 moderate grade in OP) as well as signs of inflammation reaction, such as lymphocyte count (IP vs. OP: 958.4 ± 753.7 vs. 944.8 ± 237.8/μL) and serum CRP level (IP vs. OP: 4.91 ± 6.37 vs. 3.26 ± 2.89 mg/dL). The number of current smokers and mean body mass index (BMI) were also similar between the groups. Regarding the selection of therapeutic agents, there was no significant difference in the use of dexamethasone between the two groups; however, the antiviral agent was administered more frequently in the OP group (30%) than in the IP group (0%). According to the referral letters from other hospitals, favipiravir was administered to all three patients who received the antiviral agents in the OP group.

**Fig. 2:**
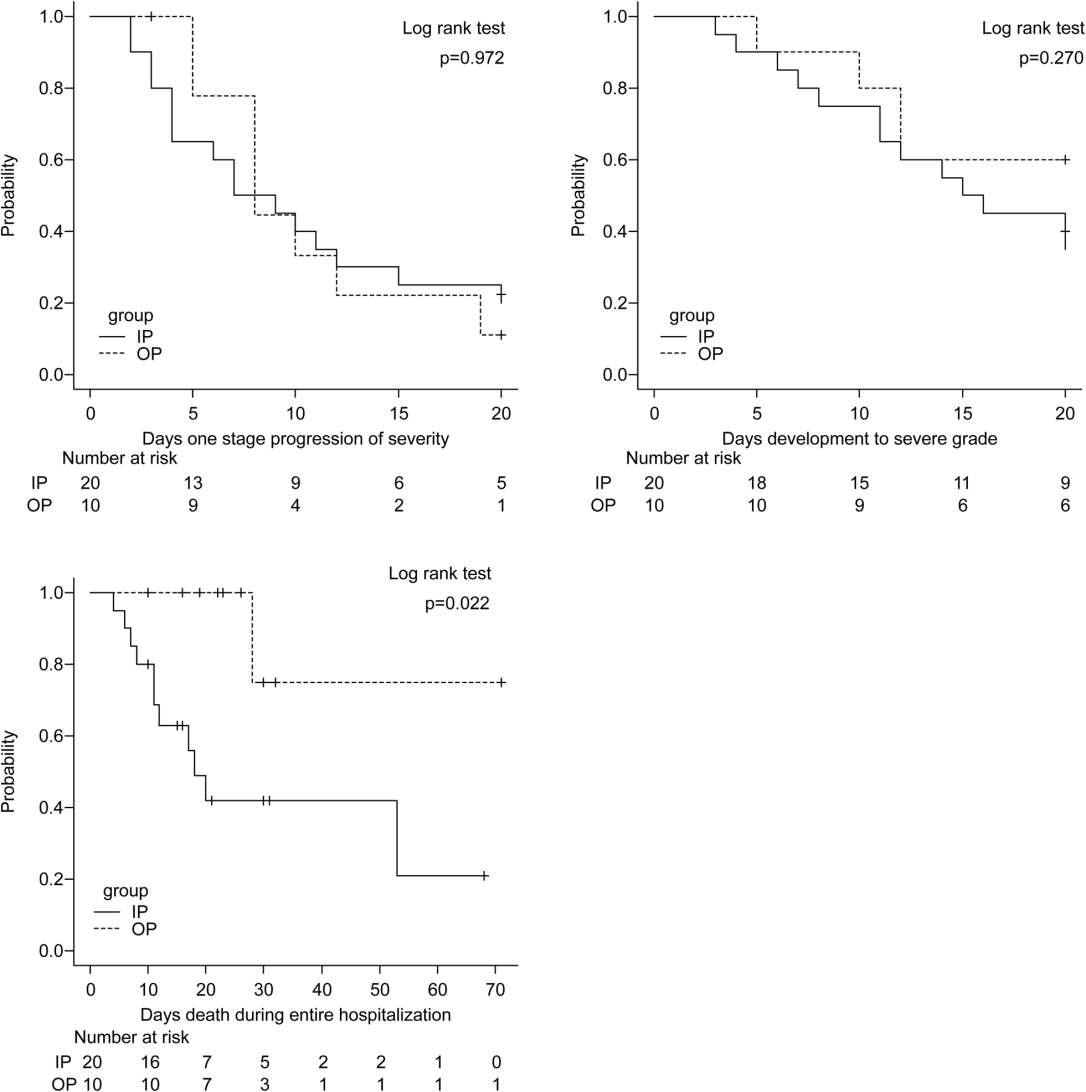
Survival analysis using Kaplan–Meier survival curves. We used Kaplan–Meier survival curves to analyze outcomes of inpatients and outpatients during the hospitalization period. We assessed the occurrence rates of the **a** one-step progression of severity, **b** development to severe grade, and **c** death during the entire hospitalization. We performed a Log-rank test, and differences were considered significant when *p* < 0.05. IP, inpatient group; OP, outpatient group

Figure 2 shows the results of the Kaplan–Meier analysis of the survival rate during the hospitalization period. Although the occurrence of one-stage progression of severity and development of severe grade did not show statistically significant differences between the OP and IP groups (Fig. 2a, b), the IP group had worse survival rates in mortality (Fig. 2c) (log-rank *p* = 0.022) than the OP group.

In the univariate analysis, using the Cox proportional hazards regression to assess the relationship between mortality and major covariates (Table 2), the IP group tended to be associated with mortality, but not significantly (HR: 7.654, 95% CI 0.983–59.580). As a significantly harmful factor, wheelchair users or certified caregivers were termed as risk factors for mortality (HR: 7.663, 95% CI 1.974–29.750); serum albumin levels had a negative association with mortality as a beneficial parameter (HR: 0.275, 95% CI 0.093–0.726). A CFS of 7 showed a trend toward a reduced risk of death, although the difference was not significant (HR 0.10, 95% CI 0.01–1.05). Other representative variates, such as age (HR 1.030, 95% CI 0.989–1.083), BMI (HR 0.958, 95% CI 0.855–1.043), and CRP levels (HR 1.080, 95% CI 0.972–1.180), had no significant correlation with mortality. Moreover, we observed no association of mortality with vital signs at diagnosis or steroid administration during hospitalization. The univariate model defined by the antiviral agent was not adopted as the 95% CI was infinite.

**Table 2:** Univariate analysis using Cox proportional hazards model for the identification of risk factors for mortality in all hemodialysis patients with COVID-19

Parameters	Hazard ratio (95% CI)	*p* value
Age (/1 year)	1.030 (0.989–1.083)	0.200
Sex (male)	1.485 (0.323–6.816)	0.611
Duration of dialysis (/1 month)	0.995 (0.984–1.005)	0.296
DM history (yes)	2.246 (0.606–8.321)	0.150
CVD history (yes)	1.924 (0.415–8.915)	0.403
Wheelchair user or certified caregiver (yes)	7.663 (1.974–29.75)	0.003
Clinical frailty score 7 (versus score 8)	0.100 (0.010–1.049)	0.059
Past smoking (yes)	0.871 (0.253–3.007)	0.828
Systolic blood pressure (/10 mmHg)	0.909 (0.709–1.140)	0.427
Diastolic blood pressure (/10 mmHg)	0.860 (0.582–1.181)	0.397
Heart rate (/1 bpm)	0.973 (0.936–1.028)	0.155
Body temperature (/ 1°)	0.557 (0.283–1.104)	0.088
SpO2 (/1%)	0.899 (0.793–1.053)	0.126
Body mass index (/1%)	0.958 (0.855–1.043)	0.429
Albumin (/1 g/dL)	0.275 (0.093–0.726)	0.012
LDH (/100 mg/dL)	0.766 (0.362–1.466)	0.446
CRP (/1 mg/dL)	1.080 (0.972–1.180)	0.108
Steroids (yes)	0.319 (0.085–1.194)	0.090
IP group (versus OP group)	7.654 (0.983–59.580)	0.052

*CI* confidence interval, *CRP* C-reactive protein, *CVD* cardiovascular disease, *DM* diabetes mellitus, *IP* inpatients, *LDH* lactate dehydrogenase isozyme, *OP* outpatients, *RAS* renin-angiotensin system, *SpO2* peripheral oxygen saturation

In multivariate analysis (Table 3), by estimating the risk of mortality induced by nosocomial COVID-19 as a clinical predictor, the IP group showed no significant relationship with mortality in models 1 (HR: 7.427, 95% CI 0.796–69.330) and 2 (HR: 8.180, 95% CI 0.281–238.200) after adjusting for major confounders or confounders plus risk variables, as identified in previous reports. Although there was no significant correlation between mortality and age (HR 0.952, 95% CI 0.875–1.036), serum albumin level (HR 0.603, 95% CI 0.120–3.029), and CRP level (HR 1.009, 95% CI 0.905–1.125) in model 2, it showed only wheelchair user or certified caregiver to be associated with mortality independently (HR 12.630, 95% CI 1.569–98.700).

**Table 3:** Multivariate analysis using Cox proportional hazards model to assess the risk factors of being in the IP group for mortality in all hemodialysis patients with COVID-19

	Model 0		Model 1		Model 2	
Parameters	Hazard ratio (95% CI)	*p* value	Hazard ratio (95% CI)	*p* value	Hazard ratio (95% CI)	*p* value
IP group (vs. OP group)	7.654 (0.983–59.580)	0.052	7.427 (0.796–69.330)	0.079	8.180 (0.281–238.200)	0.222
Age (/1 year)	–	–	1.002 (0.952–1.055)	0.947	0.952 (0.875–1.036)	0.257
Sex (male)	–	–	1.497 (0.317–7.072)	0.610	1.586 (0.305–8.264)	0.584
Albumin (/1 g/dL)	–	–	–	–	0.603 (0.120–3.029)	0.539
CRP (/1 mg/dL)	–	–	–	–	1.009 (0.905–1.125)	0.870
Wheelchair user or certified caregiver	–	–	–	–	12.630 (1.569–98.700)	0.017

Model 0, univariate analysis with the IP group

Model 1, Model 0 adjusted for age and sex

Model 2, Model 1 adjusted for serum albumin level, CRP level, and wheelchair users and certified caregivers

*CI* confidence interval, *CRP* C-reactive protein, *CVD* cardiovascular disease, *DM* diabetes mellitus, *IP* inpatients, *LDH* lactate dehydrogenase isozyme, *OP* outpatients, *RAS* renin-angiotensin system, *SpO2* percutaneous arterial oxygen saturation

### Factors involved in a nosocomial outbreak in the dialysis unit

Nosocomial infection occurred in many wards simultaneously, and the medical worker in the dialysis unit became positive after the inpatient tested positive; examination of the entire hospital did not prove the transmission within the dialysis unit. However, after fundamentally reviewing the strategies for infection control practiced in our dialysis unit before the nosocomial outbreak, we found the following potential factors that might have been involved in the nosocomial transmission: (1) beds for inpatients and outpatients were mixed on the same floor; (2) the linen were not changed after each dialysis session; and (3) the beds for patients requiring suction were separated from the next bed with only a curtain. Hence, we took the following measures: (1–1) dividing the floor into two areas, where we placed beds for inpatients and outpatients separately, and (1–2) dividing medical staff into inpatient staff and outpatient staff for a certain period to minimize the possibility of transmission of COVID-19 by the staff; (2) changing linen after each dialysis session; and (3) collecting information of inpatients who required suction in advance, whether infected or not, and placing them in a private room. As a result, to date, no outbreaks of COVID-19 have occurred in the dialysis unit, except for isolated cases due to community-acquired infections.

## Discussion

The present analysis revealed that hemodialysis inpatients who had COVID-19 during hospitalization for other treatments showed high mortality during the nosocomial outbreak of COVID-19, and ADL dependency was associated with worse mortality in hemodialysis patients with COVID-19 that spread in the winter of 2020–2021.

Nosocomial outbreaks of COVID-19 in non-dialysis patients have been reported in China [16], as well as in Japan [18]; for example, a survey in Tokyo, Japan, investigating nosocomial transmission of COVID-19 showed a 27% mortality rate in hospitalized patients [19]. A recent study on dialysis patients found that bedridden patients admitted to the nosocomial infection ward had a higher SARS-CoV-2 infection rate [20]. However, no studies have compared the demographics of hemodialysis patients with COVID-19 between those during a nosocomial outbreak and in a social community, and have analyzed the risk of nosocomial COVID-19 for mortality during this period. In our cohort study, we found an extremely high mortality rate in the IP group compared with the OP group (55% vs. 10%). Moreover, survival analysis using the Kaplan–Meier method showed that the IP group had rapid development to a worse prognosis than the OP group. The elevated mortality rate in the IP group based on the Kaplan Meier survival analysis is probably due to the following factors: (1) severe pathologies requiring hospitalization in inpatients (16 patients, 80%), such as congestive respiratory failure and extreme uremia in hemodialysis initiation (6 patients, 30%), severe infectious diseases other than COVID-19 (3 patients, 15%), cardiovascular diseases (4 patients, 20%), fractures requiring temporary rest (2 patients, 10%), and severe diabetic gangrene (1 patient, 5%); (2) during the nosocomial outbreak, it was difficult to administer effective antiviral agents; this was not only because the reports that remdesivir could be safe for dialysis patients [21] and could reduce mortality [22] had not yet been published, but also because the domestic distribution of remdesivir was unstable and limited to a few medical institutions.

Current medications for COVID-19 involve two basic strategies based on an influential randomized controlled trial: suppression of viral growth using antiviral agents, and prevention of host hyperactive immune response using immune-modulating medications such as steroids and anti-rheumatic agents. Although steroids demonstrated potential in preventing the progression of COVID-19 severity in patients with moderate or higher respiratory failure [23], no other reliable essential strategies for hemodialysis patients with COVID-19 had been established by the third wave of the pandemic in the winter of 2020–2021. As a result, there was no difference in the administration of steroids between the IP and OP groups, and although the use of antiviral agent was found to be significantly more frequent in the OP group, the fact that the drug used was favipiravir, whose efficacy remains unclear, suggests that effective treatment selection was difficult.

Notably, multivariate evaluation by Cox hazard proportional analysis with adjustment for related covariates to examine the risk for mortality by being in the IP group revealed that ADL dependency was significantly associated with the risk of mortality, and being in the IP group failed to be an independent risk of mortality. Commonly, the hemodialysis population has been reported to develop a worse prognosis due to multifactorial conditions, such as malnutrition [24], chronic inflammation, and functional dependency/frailty [25–27]. In particular, when patients with end-stage CKD initiate hemodialysis, a substantial and sustained decline in functional status emerges [28]. Therefore, maintenance of ADL by avoidance of frailty is essential for protection from critical conditions, such as severe infections, as well as for the safety of long-term dialysis procedures. Although, the relationship between ADL dependency or frailty and COVID-19 mortality has been reported in non-hemodialysis [29, 30] and hemodialysis patients [15], this study is the first to demonstrate that ADL dependency is a determinant factor for mortality in hemodialysis patients, including those who accidentally had COVID-19 due to a nosocomial outbreak.

Meanwhile, previous studies found several risk factors concerning the prognosis of hemodialysis patients with COVID-19: advanced age, decreased oxygen saturation, low diastolic blood pressure on admission, and complications, in Wuhan, China [31]; age > 70 years, longer dialysis vintage, higher CRP, and male sex, in Stockholm, Sweden [32]; older age, diabetes, local community COVID-19 rates, in London, UK [33]; and older age, heart disease, and markers of frailty, in the US [15]. A recent report of dialysis patients with COVID-19 in Japan revealed that older age (> 70 years), higher BMI, higher CRP level, and lower serum albumin levels, were aggravating factors of mortality, and that the use of remdesivir was predictive of improved mortality [22]. Older age has been identified as a common risk factor for mortality in many studies; however, we found that ADL dependency showed an influence on mortality whereas age did not, probably because of the small cohort size and absence of correlation between old age and decreased ADL. This latter phenomenon is caused by the dissociation between chronological aging and premature aging that frequently occurs in CKD [34], and is considered to be one aspect of dialysis patients whose prognosis is difficult to predict only by chronological aging. In our analysis to evaluate the relationship between age and CVD history or dialysis vintage, both of which are recognized to cause a decline in ADL, we found that there was no significant difference between age and CVD event (with CVD vs. without CVD: 69.4 ± 18.0 years vs. 64.8 ± 16.5 years), and there was no correlation on regression analysis between age and dialysis vintage (R^2^ = 0.02, *p* = 0.68). Moreover, other common risk factors for mortality reported previously, such as unstable vital status (lower blood pressure and decreased oxygen saturation) and abnormalities in serum parameters (albumin level, ferritin level, and lymphocyte count) were not identified as adverse predictors of mortality in our analysis either. This might have been because the IP group had many positive COVID-19 tests performed for screening purposes and not only after symptoms appeared, and therefore it may have been too early to evaluate the clinicopathology of COVID-19 by patient data and vital signs at the time of positive COVID-19 testing. On the other hand, various factors for mortality in dialysis patients with a diagnosis of COVID-19 have been identified to date, although this is probably influenced by the available treatment options or virus variants. Therefore, the generalization of our results to other populations infected in other waves of the COVID-19 pandemic may be challenging.

The extremely high mortality rate identified in this study emphasizes the need to take strict infection control measures for COVID-19 transmission. A previous report showed that the robust control of healthcare-associated COVID-19 could minimize further transmission of COVID-19 in local communities, as well as in the hospital [35]. Moreover, COVID-19 transmission in the dialysis unit has become a major issue not only because of the high infectivity of COVID-19 and the close space in the unit, but also because of the presence of asymptomatic infected individuals undergoing hemodialysis (18.4–21% in China [12, 36]). In addition, a study demonstrated that the dialysis unit size, the number of available private rooms, and the introduction of mask policies for asymptomatic patients, were inversely associated with positive tests for suspected COVID-19 [32]. In our dialysis unit we reviewed previous strategies for infection control and identified potential factors that might have been involved in nosocomial transmission. By taking the best possible measures, the transmission of the virus in the dialysis unit did not re-occur at the time of submission. We will regularly review whether the current strategy is optimal against the threat of viruses that might mutate in the future.

There are some limitations to this study. The first is that there may have been limitations in the criteria for assessing ADL dependency. Accumulating studies have shown the representative tools available to assess ADL dependency/frailty [37–41]. Commonly, frailty is associated with worse patient outcomes in any inpatient treatment, including medical, surgical, and intensive care [42]. In the UK, the National Institute for Clinical Excellence-released rapid COVID-19 guidelines recommends the use of the clinical frailty score (CFS) [17] for patients admitted to the intensive care unit, and landmark literature has shown a significant relationship between mortality and frailty in inpatients suffering from COVID-19 using the CFS [43]. In the present study, ADL dependency was assessed retrospectively using the CFS. However, the fact that a high CFS was not an independent factor contributing to death, despite the effectiveness of less objective parameters of wheelchair and caregiver in predicting death, suggests that there may have been limitations to the CFS’s ability to assess ADL dependency of dialysis patients with nosocomial infection, even though the number of cases is absolutely insufficient. Hence, at present, although a suitable index or scoring tools remain debatable, it would be necessary to unify the method of more detailed information collection to evaluate ADL dependency/frailty for inpatients with COVID-19, including nosocomial inpatients. Thorough and detailed collection of patient data about physical functions will help physicians decide whether to include physical therapy as an essential management strategy for frail inpatients with COVID-19. Second, we cannot ignore the difference in virus variants and vaccine progress. Although the third-wave pandemic in Japan was widespread at that time, pandemics by subsequent virus variants were even more widespread, thus leading to more infected individuals. However, probably because the older population was vaccinated, the risk of mortality decreased. In fact, a previous study showed that the mortality of COVID-19 varied depending on the pandemic phases: the mortality increased from March to April 2020, and decreased significantly in November 2020 in the US [44]. Therefore, it is uncertain whether ADL dependency detected as the risk factor for mortality in our analysis could be a risk factor for mortality associated with changing virus variants. We speculate that the risk of mortality needs to be reinvestigated each time based on the infection situation and medical system, such as vaccination and supply of available medication. Third, this retrospective cohort analysis included a small number of participants from a single center. Although the vulnerability of infected patients to nosocomial outbreaks might differ depending on the background pathologies, the risk of progression of COVID-19 in each background pathology requiring hospitalization could not be assessed due to the large disparity and small number of cases in each pathology. Hence, the generalization of our results to other inpatient populations may be challenging due to differences in scale and characteristics of medical institutions, patient comorbidities, and care protocols for hospitalized pathologies. Further studies are therefore needed to determine risk factors for mortality in nosocomial and community-acquired infections of COVID-19.

## Conclusions

We revealed that the nosocomial outbreak of COVID-19 more frequently and rapidly deteriorated the mortality of nosocomial-infected inpatients undergoing hemodialysis than that of outpatients with COVID-19 undergoing hemodialysis. Moreover, ADL dependency was an independent risk factor for mortality in hemodialysis patients, including inpatients with nosocomial COVID-19. Our findings provide some insights into the underlying pathology causing worse mortality in inpatients with COVID-19 undergoing hemodialysis. Further studies are needed to evaluate and to manage the risks of mortality caused by novel virus infections. Finally, we hope that the disclosure of this experience of nosocomial outbreak will emphasize the vulnerabilities of dialysis patients with ADL dependency, as well as the importance of robust infection control measures.

## Data Availability

The datasets used and/or analyzed during the current study are available from the corresponding author on reasonable request.

## Abbreviations

ADL: Activity of daily life
BMI: Body mass index
CFS: Clinical frailty score
CI: Confidence interval
CKD: Chronic kidney disease
COVID-19: Coronavirus disease 2019
CRP: C-reactive protein
CVD: Cardiovascular disease
HR: Hazard ratio
ICU: Intensive care unit
IP: Inpatient
OP: Outpatient
SARS-CoV-2: Severe acute respiratory syndrome coronavirus 2
SpO2: Peripheral oxygen saturation
